# 4D-Dynamic Representation of DNA/RNA Sequences: Studies on Genetic Diversity of *Echinococcus multilocularis* in Red Foxes in Poland

**DOI:** 10.3390/life12060877

**Published:** 2022-06-10

**Authors:** Dorota Bielińska-Wąż, Piotr Wąż, Anna Lass, Jacek Karamon

**Affiliations:** 1 Department of Radiological Informatics and Statistics, Medical University of Gdańsk, 80-210 Gdańsk, Poland; 2 Department of Nuclear Medicine, Medical University of Gdańsk, 80-210 Gdańsk, Poland; phwaz@gumed.edu.pl; 3 Department of Tropical Parasitology, Medical University of Gdańsk, 81-519 Gdynia, Poland; anna.lass@gumed.edu.pl; 4 Department of Parasitology and Invasive Diseases, National Veterinary Research Institute, 24-100 Puławy, Poland; j.karamon@piwet.pulawy.pl

**Keywords:** data analysis, bioinformatics, alignment-free methods, moments of inertia

## Abstract

The 4D-Dynamic Representation of DNA/RNA Sequences, an alignment-free bioinformatics method recently developed by us, has been used to study the genetic diversity of *Echinococcus multilocularis* in red foxes in Poland. Sequences of three mitochondrial genes, i.e., NADH dehydrogenase subunit 2 (*nad2*), cytochrome b (*cob*), and cytochrome c oxidase subunit 1 (*cox1*), are analyzed. The sequences are represented by sets of material points in a 4D space, i.e., 4D-dynamic graphs. As a visualization of the sequences, projections of the graphs into 3D space are shown. The differences between 3D graphs corresponding to European, Asian, and American haplotypes are small. Numerical characteristics (*sequence descriptors*) applied in the studies can recognize the differences. The concept of creating descriptors of 4D-dynamic graphs has been borrowed from classical dynamics; these are coordinates of the centers or mass and moments of inertia of 4D-dynamic graphs. Based on these descriptors, classification maps are constructed. The concentrations of points in the maps indicate one Polish haplotype (EmPL9) of Asian origin.

## 1. Introduction

Recently, a rapid growth of the experimental data in nucleotide databases can be observed, which stimulated the development of mathematical methods to describe these large and complex objects. One group of approaches is formed by the so-called alignment-free bioinformatics methods. For reviews of alignment-free methods, see [1,2]. They are an alternative to standard, alignment-based sequence analysis approaches, e.g., ClustalW [3], Blast [4], Needleman–Wunsch algorithm [5], or T-Coffee [6]. Alignment-free methods are usually computationally simple and there are no sequence limitations. They are particularly useful for Big Data analysis and research on various aspects of similarity between the biological (DNA, RNA, protein) sequences.

Similarity of complex objects is not unique. Multi-dimensional objects can be similar in one aspect/property and very different if other characteristics are taken into account. Different aspects of similarity may be relevant to different problems. Let us take a model example and consider two different pairs of DNA sequences:

1. G G T T
  G G A A
2. G T G T
  G A G A

In both cases, the similarity value is 50%, but non-zero contributions to the final result come from different positions of G in the sequences. In the first case, G are cumulative at the beginning of the sequences, and in the second one the distributions of G are symmetric. The same results are also obtained if, for example, G is replaced by C. Different structures give the same result in standard alignment methods. The degree of non-uniqueness increases with the lengths of the sequences. Advantages of non-standard (alignment-free) methods include the suitability for Big Data analysis with no restrictions for the sequences, as already mentioned, as well as a variety of derived information. In non-standard methods, we obtain a series of values characterizing different properties of a single sequence. The similarity of these properties can be studied separately using non-standard methods and may be correlated with different biological consequences. Therefore, the creation of new methods is very important to reveal some hidden properties of the sequences.

Similarity/dissimilarity analysis is strictly related to classification studies, which is an interdisciplinary problem [7,8]. For example, we obtained information about different types of objects by examining their similarity in the quality of life research [9,10], or in bioinformatics [11,12].

In bioinformatics, there are many different alignment-free methods. For example, Zhou et al. constructed a complex network for similarity/dissimilarity analysis of DNA sequences [13]. We represented the protein sequence as a set of material points in a 20D space [14]. Saw et al. analyzed the similarity of DNA sequences using the fuzzy integral with a Markov chain [15]. Lichtblau applied frequency chaos game representation and signal processing for genomic sequence comparison [16]. He et al. introduced a numerical representation of a DNA sequence, called the Subsequence Natural Vector, and applied it for HIV-1 subtype classification [17].

A subgroup within *alignment-free* bioinformatics methods is formed by the so-called *Graphical Representations of Biological Sequences*, applicable to both graphical and numerical similarity/dissimilarity analysis of biological sequences. It is not obvious how to represent graphically multidimensional objects in two or three dimensions to reveal the most important features without losing information. A variety of approaches have been developed, bringing together ideas from different fields of science, and each of them focuses on various aspects of similarity. Method names are often associated with some properties or ideas applied to the construction of graphs or numerical characteristics describing the graphs. The first graphical representation methods were based on *walks* in three [18,19] and two [20,21,22] dimensions. Since then, there has been a dynamic development of the graphical bioinformatics branch observed (for reviews see [23,24]). Let us just mention the last few methods of graphical representation: in the “Spider representation of DNA sequences”, the graphs resemble a spider’s web [25]; in a method called by us “Spectral-dynamic representation of DNA sequences”, the plots resemble atomic, molecular, or stellar spectra composed of sequences of sharp spectral lines [11]. For the numerical characterization of these plots, we applied some ideas used in classical dynamics. Hu et al. applied fractal interpolation in their graphical representation of protein sequences [26]. Graphical representations of protein sequences based on physiochemical properties may be found in works by Mahmoodi-Reihani et al. [27], or by Xie and Zhao [28]. A graphical representation of DNA sequences proposed by Xie et al. is based on trigonometric functions [29]. The 2D graphic representation of the DNA sequence proposed by Liu is based on the horizon lines [30]. Another 2D graphical representation of DNA sequences proposed by Wu et al. is based on variant map [31]. The goal is to create approaches in which both graphs and numerical characteristics, often referred to as *sequence descriptors*, represent a biological sequence in a unique (i.e., *degeneracy-free*) way. The first sequence descriptors related to graphical representation of sequences were designed by Raychaudhury and Nandy [32] and by Randić et al. [33]. Since then, many approaches have been created, e.g., spectral moments in the sequence similarity studies were considered by Agüero-Chapin et al. [34] (for review see [35]).

In the present work, we apply 4D-Dynamic Representations of DNA/RNA Sequences created by us [12]. This is a multidimensional alignment-free bioinformatics method, but it also offers some kind of visualization (for details see subsequent section). We applied this method for a characterization of SARS-CoV-2 and Zika viruses. In the present work, we perform analogous studies on genetic diversity of *Echinococcus multilocularis* in red foxes in Poland. Alveolar echinococcosis is a serious parasitic zoonosis caused by *Echinococcus multilocularis*, Leuckart 1863. E. multilocularis was found in Poland in relatively high percentages in red foxes; in some regions, the prevalence reached up to approximately 50% [36]. More than one hundred cases of human alveolar echinococcosis were described before 2013 [37]. The present study is a continuation of our previous work in which the results have been obtained using a standard ClustalW method [38].

## 2. Materials and Methods

In the present studies, we apply the 4D-Dynamic Representation of DNA/RNA Sequences—an alignment-free bioinformatics method proposed by us [12]. In this approach, the DNA/RNA sequence is represented as a set of material points in a 4D space, called the *4D-dynamic graph*. The distribution of the points in the space is characteristic for the sequence. A 4D-dynamic graph is created using a method of shifts (*walk*) starting from the point with coordinates (0,0,0,0). The first shift is performed according to the unit vector representing the first nucleobase in the sequence. Starting from the end of this vector, the second shift is performed according to the unit vector representing the second nucleobase in the sequence. The process continues until the last nucleobase in the sequence. At the end of each vector, a material point is located with the mass $m_{i}=1$. Then, the total mass of the 4D-dynamic graph is the length of the sequence (*N*):

(1) $$N=\sum_{i=1}^{N}m_{i}.$$

We represent the nucleobases by the following unit vectors: adenine by the vector A = (1,0,0,0), cytosine by C = (0,1,0,0), guanine by G = (0,0,1,0), and thymine/uracil by T/U = (0,0,0,1). The final similarity relations between the sequences are the same for different assignments of particular unit vectors to the nucleobases. Choosing the mass different from 1, the final relative similarity relations also remain the same. The mass of each material point and the unit vectors representing particular nucleobases should be the same for all the sequences.

An example of the construction of the 4D-dynamic graph for a model sequence AUGAC is given in [12].

As a visualization of the 4D-dynamic graphs, we apply their projections into 2D or 3D spaces. For example, if we put $x_{i}^{1}$ and $x_{i}^{2}$ coordinates equal to zero, then we obtain a 2D projection, i.e., $x^{3}x^{4}$-graph. The distributions of the material points in the 3D or 2D spaces give some information about the locations of three or two nucleobases along the sequences.

As the numerical characteristics of the 4D-dynamic graphs (*sequence descriptors*), we apply values analogous to the ones used in the classical dynamics. One kind of such sequence descriptors are the coordinates of the center of mass of the 4D-dynamic graph:

(2) $$\mu^{k}=\frac{\sum_{i=1}^{N}m_{i}x_{i}^{k}}{\sum_{i=1}^{N}m_{i}}=\frac{1}{N}\sum_{i=1}^{N}x_{i}^{k}.$$

$x_{i}^{k}$ are the coordinates of the mass $m_{i}$ in the 4D space and k=1,2,3,4.

Another kind of value analogous to the one used in the classical dynamics is the tensor of the moment of inertia of 4D-dynamic graph. It is given by the matrix

(3) $$\hat{I}=\left(\begin{array}{cccc} I_{1\;1} & I_{1\;2} & I_{1\;3} & I_{1\;4}\\ I_{2\;1} & I_{2\;2} & I_{2\;3} & I_{2\;4}\\ I_{3\;1} & I_{3\;2} & I_{3\;3} & I_{3\;4}\\ I_{4\;1} & I_{4\;2} & I_{4\;3} & I_{4\;4} \end{array}\right)$$

with the elements:

(4) $$I_{j\;j}=\sum_{i=1}^{N}m_{i}\sum_{k=1}^{4}{\left[{\hat{x}}_{i}^{k}\left(1-\delta_{jk}\right)\right]}^{2},$$

(5) $$I_{j\;k}=I_{k\;j}=-\sum_{i=1}^{N}m_{i}{\hat{x}}_{i}^{j}{\hat{x}}_{i}^{k},$$

where

$$\delta_{jk}=\left\{\begin{array}{cc} 1 & j=k,\\ 0 & j\neq k \end{array}\right.$$

is the Kronecker delta. ${\hat{x}}_{i}^{k}$ are the coordinates of $m_{i}$ in the Cartesian coordinate system for which the origin has been selected at the center of mass:

(6) $${\hat{x}}_{i}^{k}=x_{i}^{k}-\mu^{k}.$$

The eigenvalue problem of the tensor of inertia is defined as:

(7) $$\hat{I}\omega_{k}=I_{k}\omega_{k},\;\;\;k=1,2,3,4,$$

where $I_{k}$ are the eigenvalues and $\omega_{k}$ are the eigenvectors. The eigenvalues are obtained by solving the fourth-order secular equation:

(8) $$\det(\hat{I}-I\hat{E})=0,$$

where $\hat{E}$ is 4×4 unit matrix. The eigenvalues $I_{k}$ are called the *principal moments of inertia*.

As the sequence descriptors, we apply the normalized principal moments of inertia:

(9) $$r_{k}^{4D}=\sqrt{\frac{I_{k}}{N}},\;\;\;k=1,2,3,4.$$

The presented method is applied to estimate the genetic diversity of the cestode *Echinococcus multilocularis*, Leuckart 1863, in Poland based on sequence analysis of the mitochondrial genes of worms isolated using the sedimentation and counting technique [39] from the intestines of red foxes *Vulpes vulpes* (Linnaeus). More details concerning the isolation of parasites, sample preparation, polymerase chain reactions (PCRs), and sequencing were described earlier [38]. The nucleotide sequence data used for the calculations are available in GenBank. Sequences of three mitochondrial genes, i.e., NADH dehydrogenase subunit 2 (*nad2*), cytochrome b (*cob*), and cytochrome c oxidase subunit 1 (*cox1*), are analyzed (for the accession numbers see subsequent section) [38,40].

## 3. Results and Discussion

**Figure 1:**
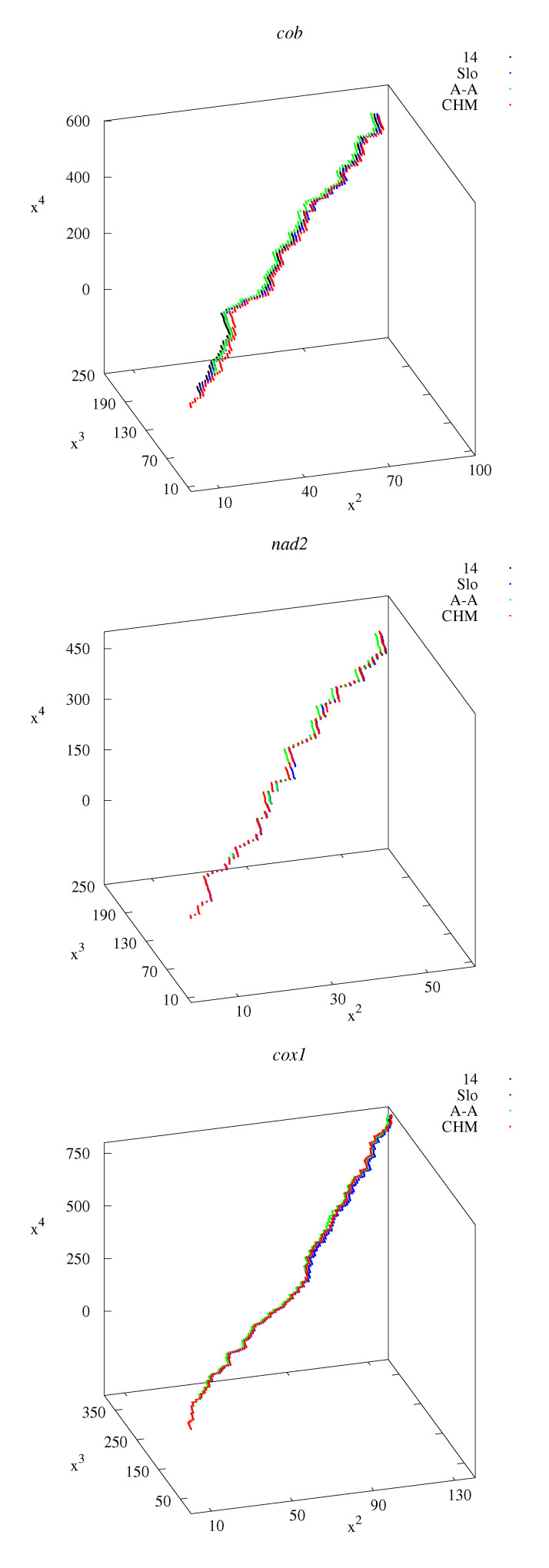
$x^{2}x^{3}x^{4}$-graphs representing *cob* (**top** panel), *nad2* (**middle** panel), and *cox1* (**bottom** panel) genes. Notations: 14—Polish haplotype (sequences No. 14 in Table 1, Table 2 and Table 3); Slo—Slovakia; A-A—USA, Alaska (St. Lawrence Island); CHM—China (Inner Mongolia).

Figure 1 shows examples of projections of the 4D-dynamic graphs to 3D space: $x^{2}x^{3}x^{4}$-graphs. The differences between the graphs representing the sequences for different countries (Poland, Slovakia, USA, China) are small. The corresponding principal moments of inertia for all the sequences used in the calculations are shown in Table 1, Table 2, Table 3, Table 4, Table 5 and Table 6.

In our previous work, combined sequence analysis of three genes (*cob*, *nad2*, *cox1*) exhibited fifteen Polish haplotypes (EmPL1–EmPL15). Separate analyzes within individual genes showed less differentiation. The number of haplotypes is smaller for *cob*, *nad2*, and *cox1* genes. They are denoted by the letters A-J (see Table 1, Table 2 and Table 3) [38]. As a consequence, in some cases, the sequence descriptors are the same. For example, the descriptors of sequences No. 1 and No. 7 in Table 3 (haplotytypes A) are the same. All the values for particular genes are similar. For example, the principal moments of inertia are similar for sequences No. 6 (EmPL6 cox_C) and No. 7 (EmPL7 cox_A) (Table 3). They are equal to 117.0, 117.0, 116.9, and 131.1 for sequence No. 6 and to 117.1, 117.1, 117.0, and 132.2 for sequence No. 7.

**Figure 2:**
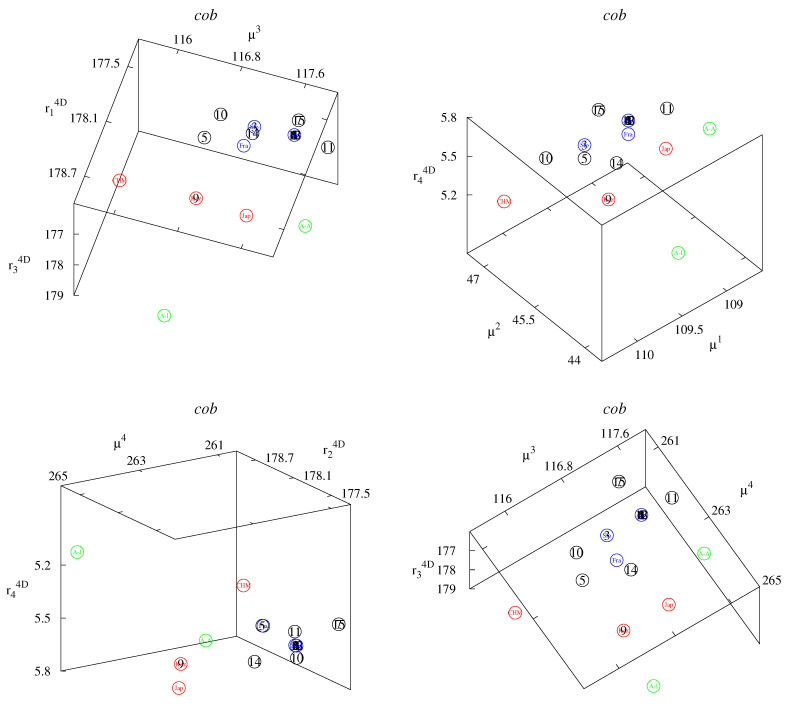
Classification maps for *cob* gene: $r_{k}^{4D}-r_{l}^{4D}-\mu^{m}$ (**left** panel) and $\mu^{k}-\mu^{l}-r_{m}^{4D}$ (**right** panel); k,l,m=1,2,3,4. Colors: blue—Europe excluding Poland; red—Asia; green—America; black—Poland. Detailed notations: 1,2,…15—Polish haplotypes (Table 1); A-A—USA, Alaska (St. Lawrence Island); A-I—USA, Indiana; Aus—Austria; CHM—China (Inner Mongolia); Fra—France; Jap—Japan (Hokkaido); Kaz—Kazakhstan; Slo—Slovakia.

**Figure 3:**
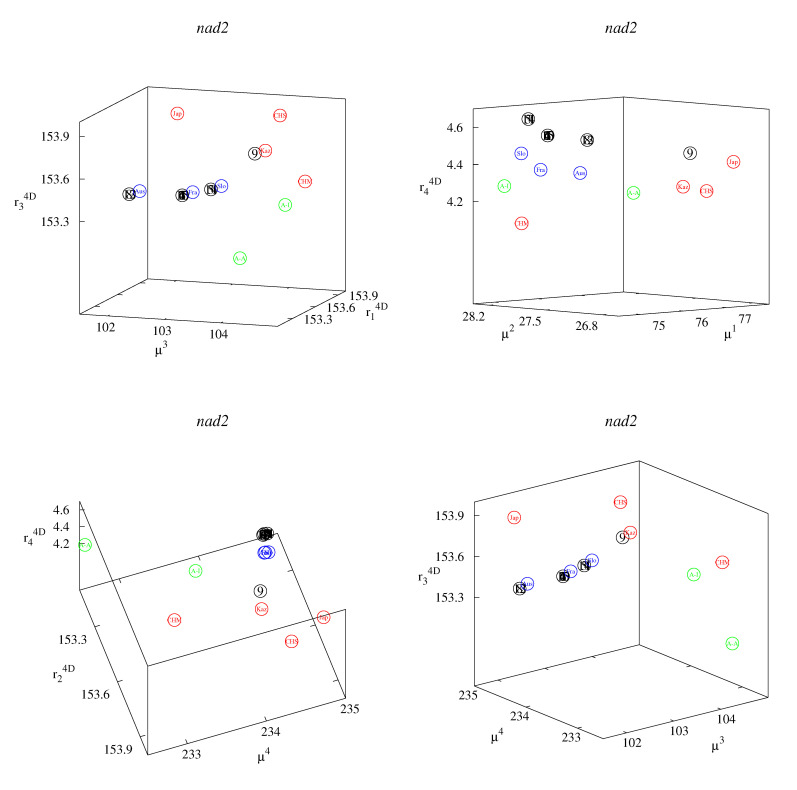
Classification maps for *nad2* gene: $r_{k}^{4D}-r_{l}^{4D}-\mu^{m}$ (**left** panel) and $\mu^{k}-\mu^{l}-r_{m}^{4D}$ (**right** panel); k,l,m=1,2,3,4. Colors: blue—Europe excluding Poland; red—Asia; green—America; black—Poland. Detailed notations: 1,2,…15—Polish haplotypes (Table 2); A-A—USA, Alaska (St. Lawrence Island); A-I—USA (Indiana); Aus—Austria; CHM—China (Inner Mongolia); CHS—China (Sichuan); Fra—France; Jap—Japan (Hokkaido); Kaz—Kazakhstan; Slo—Slovakia.

**Figure 4:**
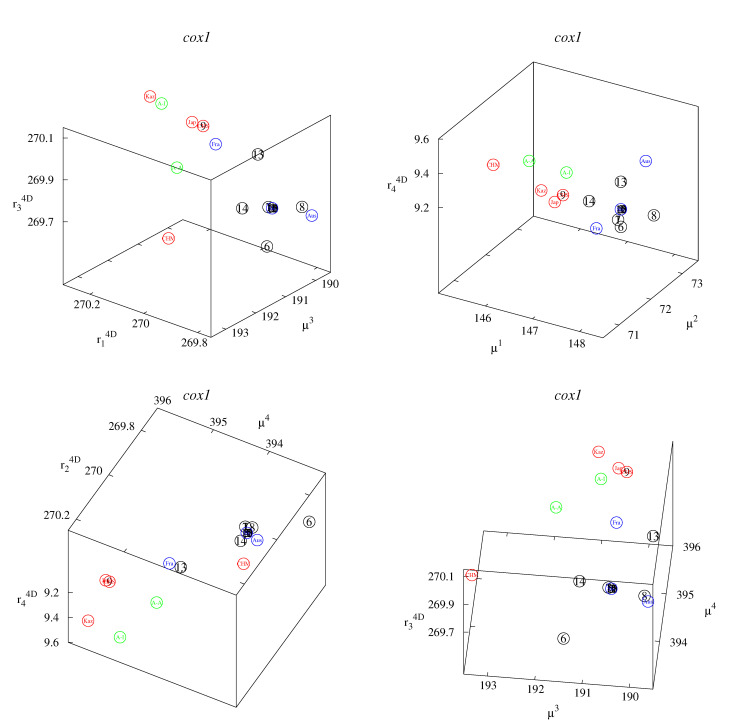
Classification maps for the *cox1* gene: $r_{k}^{4D}-r_{l}^{4D}-\mu^{m}$ (**left** panel) and $\mu^{k}-\mu^{l}-r_{m}^{4D}$ (**right** panel); k,l,m=1,2,3,4. Colors: blue—Europe excluding Poland; red—Asia; green—America; black—Poland. Detailed notations: 1,2,…15—Polish haplotypes (Table 3); A-A—USA, Alaska (St. Lawrence Island); A-I—USA, Indiana; Aus—Austria; CHM—China (Inner Mongolia); CHS—China (Sichuan); Fra—France; Jap—Japan (Hokkaido); Kaz—Kazakhstan; Slo—Slovakia.

**Figure 5:**
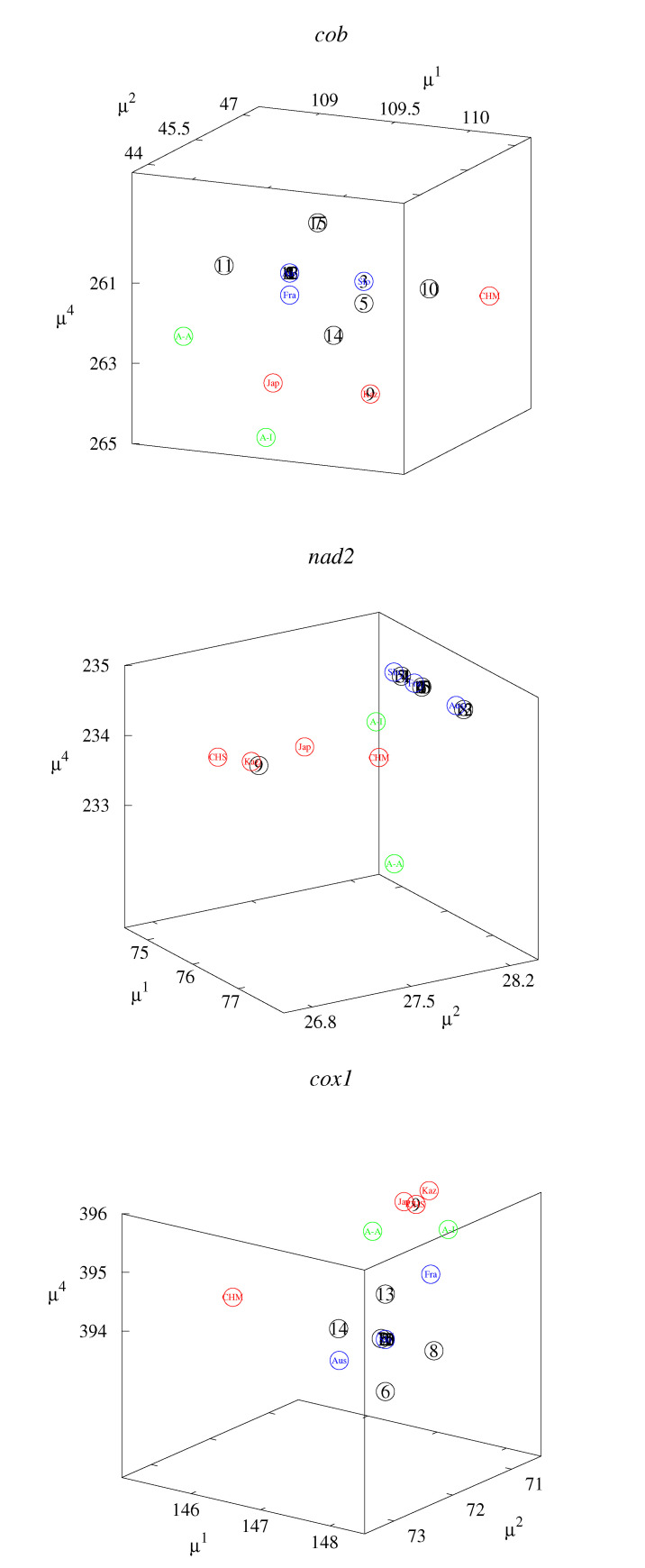
Classification maps $\mu^{1}-\mu^{2}-\mu^{4}$ for *cob* (**top** panel), *nad2* (**middle** panel), and *cox1* (**bottom** panel) genes. The colors and the detailed notations are the same as in Figure 2, Figure 3 and Figure 4.

**Figure 6:**
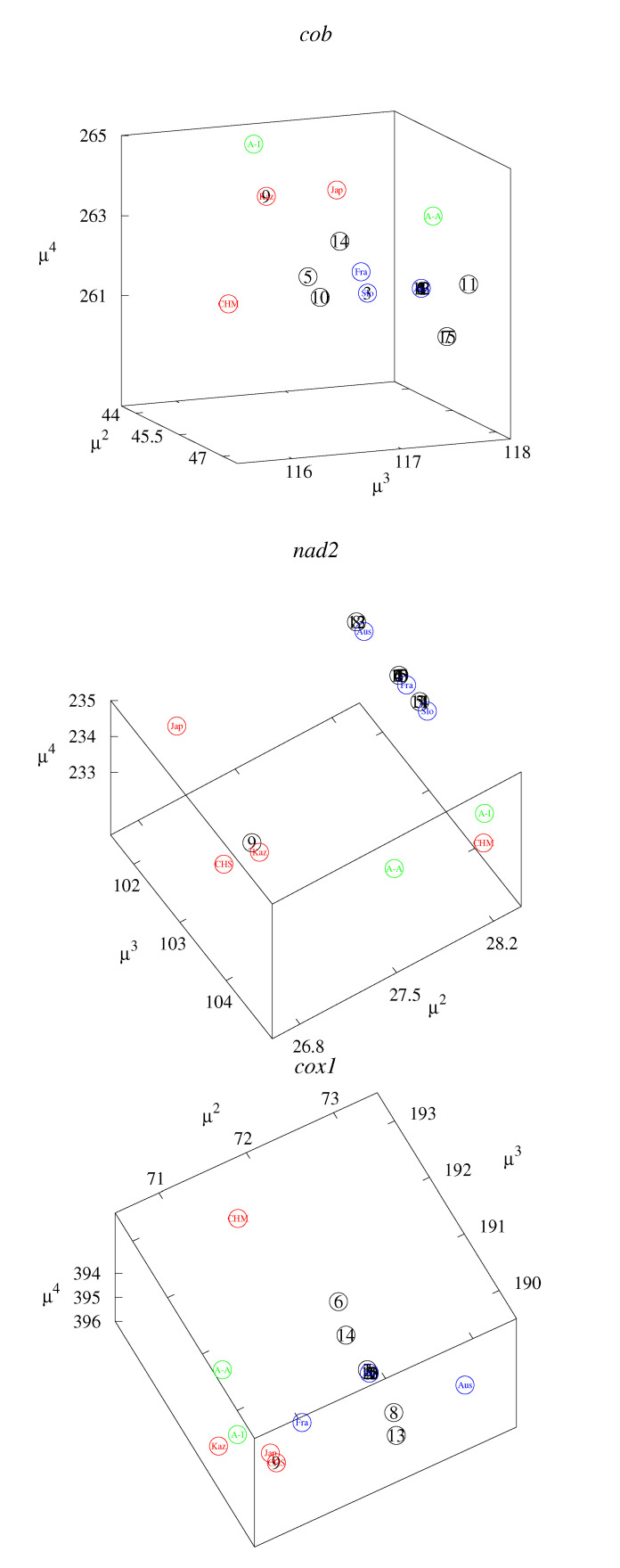
Classification maps $\mu^{2}-\mu^{3}-\mu^{4}$ for *cob* (**top** panel), *nad2* (**middle** panel), and *cox1* (**bottom** panel) genes. The colors and the detailed notations are the same as in Figure 2, Figure 3 and Figure 4.

These small differences can be better observed in the classification maps (Figure 2, Figure 3, Figure 4, Figure 5 and Figure 6). Figure 2, Figure 3 and Figure 4 show $r_{k}^{4D}-r_{l}^{4D}-\mu^{m}$ and $\mu^{k}-\mu^{l}-r_{m}^{4D}$ classification maps. Figure 2 represents the *cob* gene, Figure 3 represents the *nad2* gene, and Figure 4 represents the *cox1* gene. Figure 5 shows $\mu^{1}-\mu^{2}-\mu^{4}$ classification maps for all three genes. Figure 6 shows $\mu^{2}-\mu^{3}-\mu^{4}$ also for all three genes. The points in the maps corresponding to fourteen Polish haplotypes EmPL1, EmPL2, *…* EmPL8 and EmPL10, EmPL11, *…* EmPL15 are concentrated close to the ones representing European clades. Several Polish haplotypes nearly overlap with some European clades (for example with Austria in Figure 2 or with Slovakia in Figure 4). The exception is the Polish haplotype EmPL9. The points representing this sequence are concentrated close to the points representing Asian clades. In particular, Kazakhstan is the closest point to EmPL9 in: Figure 2 (all panels), Figure 3 (all panels), Figure 5 (panels top, middle), and Figure 6 (panels top, middle). This means that the largest similarities between EMPL9 and Kazakhstan are observed for *cob* and *nad2* genes in all the aspects considered. Figure 4, Figure 5 (bottom panel), and Figure 6 (bottom panel) show the classification maps for the *cox1* gene. In these cases, China (Sichuan) and Japan (Hokkaido) are the closest points to EMPL9.

**Figure 7:**
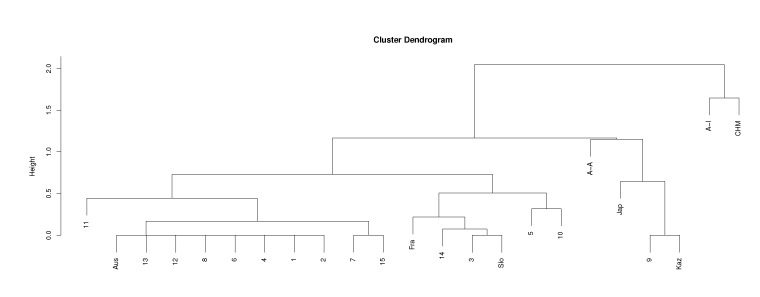
Cluster dendrogram obtained using Euclidean distance measure and $r_{3}^{4D}$, $r_{1}^{4D}$, and $\mu^{3}$ for the *cob* gene (top left panel of Figure 2).

The results coming from our method can be also presented in a form similar to phylogenetic trees of the standard methods. Figure 7 shows cluster dendrogram for the *cob* gene using $r_{3}^{4D}$, $r_{1}^{4D}$, $\mu^{3}$, and the Euclidean distance measure. This dendrogram is another representation of the results of the calculations shown in the top left panel of Figure 2.

**Figure 8:**
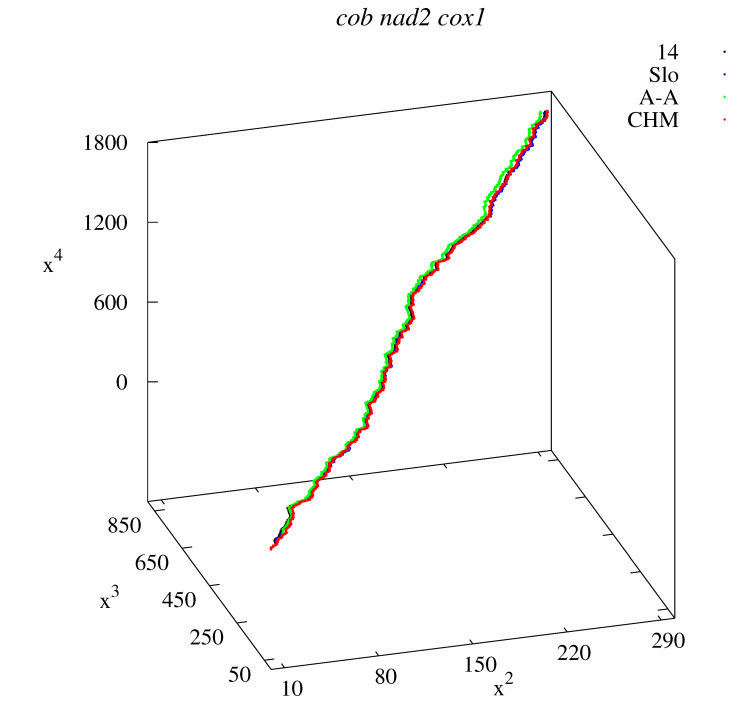
$x^{2}x^{3}x^{4}$-graphs representing *cob nad2 cox1* genes.

The method has no restriction as far as the lengths of the sequences are concerned. Within this method, it is also possible to compensate the information coming from three genes separately into one sequence. Figure 8 shows the $x^{2}x^{3}x^{4}$-graphs for combined long sequences *cob*, *nad2*, and *cox1* genes. The same four examples (14; Slo; A-A; CHM) are displayed as in Figure 1. Analogous calculations of the descriptors, as the ones shown in Figure 2, Figure 3, Figure 4, Figure 5, Figure 6 and Figure 7, can be performed for these concatenated data from the three mitochondrial genes.

## 4. Conclusions

In the present work, non-standard bioinformatics studies on the genetic diversity of the cestode *Echinococcus multilocularis* in red foxes in Poland are performed. The 4D-Dynamic Representation of DNA/RNA Sequences, an alignment-free method proposed by us, has been applied [12].

Visualization of multidimensional method is restricted, but some aspects (appropriate projections into 3D space) are shown. The sequences corresponding to European, Asian, and American haplotypes are similar to each other, so the corresponding 3D projections nearly overlap [Figure 1 all panels (sequences No. 14 in Table 1, Table 2 and Table 3; No. 3, 6, and 8 in Table 4; and No. 3, 7, and 9 in Table 5 and Table 6), and Figure 8].

We observed much larger differences for coronaviruses in our previous study [12]. Our studies have shown that the distribution of clusters of points which emerged in the classification maps supports the hypothesis that SARS-CoV-2 may have originated in bat and in pangolin [12].

The considered sequence descriptors are sensitive enough to study the differences for *Echinococcus multilocularis*. Our first report based on the standard bioinformatics method indicated one Polish haplotype (EmPL9 found only in northeast Poland) of probable Asian origin [38]. The present studies indicate aspects of similarities (descriptors related to some properties of the sequences represented in the axes of the maps), in which Polish haplotypes are similar to sequences for different countries. By analyzing the clusters of points in the classification maps (Figure 2, Figure 3, Figure 4, Figure 5 and Figure 6), the Asian origin of one Polish haplotype (EmPL9) is confirmed.

In summary, by choosing the descriptors, we can reveal different properties of the sequences. In particular, the principal moments of inertia (the values used in the classical dynamics) are equal to the moments of inertia associated with the rotations around the principal axes. The moment of inertia of an object around a rotational axis describes how difficult it is to induce the rotation of the object around this axis. If the mass is concentrated far away from the axis, it is difficult to accelerate into spinning fast and the moment of inertia is large. As a consequence, the descriptors based on moments of inertia reflect the concentrations of masses of the 4D-dynamic graphs around the axes. This way, we can compare the shapes of the graphs representing the sequences.

The correct interpretation of biological and medical data strongly depends on the accuracy of the mathematical models used. Because the accuracy of the presented method is very high (the descriptors used in this method can recognize a difference by a single nucleobase in the compared sequences) the medical importance of the presented approach is significant.

An attractive application of this approach in our future research is predicting the development of viral sequences. Building a predictive model can be crucial in dealing with the future epidemics. Pilot calculations for the Zika virus showed that such an approach could be used to describe the time evolution of the viral genome sequences [12].

## Figures and Tables

**Table 1:** Principal moments of inertia of 4D-dynamic graphs representing the *cob* gene for Poland (N=1068).

No.	Accession	Polish Haplotype	$I_{1}/10^{5}$	$I_{2}/10^{5}$	$I_{3}/10^{5}$	$I_{4}/10^{2}$
1	KY205662	EmPL1 cob_A	335.9	335.8	335.7	325.5
2	KY205663	EmPL2 cob_G	335.9	335.8	335.7	325.5
3	KY205664	EmPL3 cob_E	336.0	335.9	335.8	325.7
4	KY205665	EmPL4 cob_A	335.9	335.8	335.7	325.5
5	KY205666	EmPL5 cob_D	336.7	336.6	336.5	313.5
6	KY205667	EmPL6 cob_A	335.9	335.8	335.7	325.5
7	KY205668	EmPL7 cob_H	335.4	335.4	335.3	313.6
8	KY205669	EmPL8 cob_A	335.9	335.8	335.7	325.5
9	KY205670	EmPL9 cob_B	338.5	338.4	338.3	341.9
10	KY205671	EmPL10 cob_C	335.9	335.8	335.7	333.7
11	KY205672	EmPL11 cob_F	336.0	335.9	335.8	316.7
12	KY205673	EmPL12 cob_A	335.9	335.8	335.7	325.5
13	KY205674	EmPL13 cob_A	335.9	335.8	335.7	325.5
14	KY205675	EmPL14 cob_J	336.2	336.1	336.0	332.8
15	KY205676	EmPL15 cob_I	335.4	335.4	335.3	313.6

**Table 2:** Principal moments of inertia of 4D-dynamic graphs representing the *nad2* gene for Poland. (N=882).

No.	Accession	Polish Haplotype	$I_{1}/10^{5}$	$I_{2}/10^{5}$	$I_{3}/10^{5}$	$I_{4}/10^{2}$
1	KY205692	EmPL1 nad_A	207.7	207.7	207.6	181.5
2	KY205693	EmPL2 nad_A	207.7	207.7	207.6	181.5
3	KY205694	EmPL3 nad_D	207.8	207.8	207.7	189.3
4	KY205695	EmPL4 nad_A	207.7	207.7	207.6	181.5
5	KY205696	EmPL5 nad_D	207.8	207.8	207.7	189.3
6	KY205697	EmPL6 nad_A	207.7	207.7	207.6	181.5
7	KY205698	EmPL7 nad_A	207.7	207.7	207.6	181.5
8	KY205699	EmPL8 nad_C	207.7	207.6	207.6	178.2
9	KY205700	EmPL9 nad_B	208.4	208.4	208.3	176.9
10	KY205701	EmPL10 nad_A	207.7	207.7	207.6	181.5
11	KY205702	EmPL11 nad_D	207.8	207.8	207.7	189.3
12	KY205703	EmPL12 nad_C	207.7	207.6	207.6	178.2
13	KY205704	EmPL13 nad_C	207.7	207.6	207.6	178.2
14	KY205705	EmPL14 nad_D	207.8	207.8	207.7	189.3
15	KY205706	EmPL15 nad_A	207.7	207.7	207.6	181.5

**Table 3:** Principal moments of inertia of 4D-dynamic graphs representing the *cox1* gene for Poland. (N=1608).

No.	Accession	Polish Haplotype	$I_{1}/10^{6}$	$I_{2}/10^{6}$	$I_{3}/10^{6}$	$I_{4}/10^{3}$
1	KY205677	EmPL1 cox_A	117.1	117.1	117.0	132.2
2	KY205678	EmPL2 cox_B	117.1	117.1	117.0	134.1
3	KY205679	EmPL3 cox_B	117.1	117.1	117.0	134.1
4	KY205680	EmPL4 cox_B	117.1	117.1	117.0	134.1
5	KY205681	EmPL5 cox_B	117.1	117.1	117.0	134.1
6	KY205682	EmPL6 cox_C	117.0	117.0	116.9	131.1
7	KY205683	EmPL7 cox_A	117.1	117.1	117.0	132.2
8	KY205684	EmPL8 cox_D	117.1	117.0	117.0	134.6
9	KY205685	EmPL9 cox_E	117.3	117.3	117.2	140.4
10	KY205686	EmPL10 cox_B	117.1	117.1	117.0	134.1
11	KY205687	EmPL11 cox_B	117.1	117.1	117.0	134.1
12	KY205688	EmPL12 cox_B	117.1	117.1	117.0	134.1
13	KY205689	EmPL13 cox_F	117.2	117.2	117.1	138.9
14	KY205690	EmPL14 cox_G	117.1	117.1	117.0	134.1
15	KY205691	EmPL15 cox_B	117.1	117.1	117.0	134.1

**Table 4:** Principal moments of inertia of 4D-dynamic graphs representing the *cob* gene for different countries (N=1068).

No.	Accession	Country	$I_{1}/10^{5}$	$I_{2}/10^{5}$	$I_{3}/10^{5}$	$I_{4}/10^{2}$
1.	AB461395	Austria	335.9	335.8	335.7	325.5
2.	AB461396	France	336.6	336.5	336.4	313.2
3.	AB461397	Slovakia	336.0	335.9	335.8	325.7
4.	AB461398	Kazakhstan	338.5	338.4	338.3	341.9
5.	AB461399	Japan (Hokkaido)	338.6	338.5	338.4	359.2
6.	AB461400	USA (Alaska)	338.4	338.4	338.3	328.7
7.	AB461401	USA (Indiana)	342.1	342.1	342.0	281.8
8.	AB461402	China (Mongolia)	338.5	338.5	338.4	298.7

**Table 5:** Principal moments of inertia of 4D-dynamic graphs representing the *nad2* gene for different countries (N=882).

No.	Accession	Country	$I_{1}/10^{5}$	$I_{2}/10^{5}$	$I_{3}/10^{5}$	$I_{4}/10^{2}$
1.	AB461403	Austria	207.7	207.7	207.6	164.6
2.	AB461404	France	207.8	207.7	207.7	167.3
3.	AB461405	Slovakia	207.9	207.8	207.8	174.7
4.	AB461406	Kazakhstan	208.4	208.4	208.3	163.2
5.	AB461407	Japan (Hokkaido)	208.9	208.9	208.8	171.8
6.	AB461408	China (Sichuan)	209.0	208.9	208.8	162.1
7.	AB461409	USA (Alaska)	206.8	206.8	206.7	157.7
8.	AB461410	USA (Indiana)	207.7	207.6	207.6	161.7
9.	AB461411	China (Mongolia)	208.0	208.0	207.9	146.7

**Table 6:** Principal moments of inertia of 4D-dynamic graphs representing the *cox1* gene for different countries (N=1608).

No.	Accession	Country	$I_{1}/10^{6}$	$I_{2}/10^{6}$	$I_{3}/10^{6}$	$I_{4}/10^{3}$
1.	AB461412	Austria	117.0	117.0	116.9	139.0
2.	AB461413	France	117.3	117.3	117.2	134.3
3.	AB461414	Slovakia	117.1	117.1	117.0	134.1
4.	AB461415	Kazakhstan	117.4	117.4	117.3	142.8
5.	AB461416	Japan (Hokkaido)	117.3	117.3	117.2	138.8
6.	AB461417	China (Sichuan)	117.3	117.3	117.2	140.4
7.	AB461418	USA (Alaska)	117.3	117.3	117.2	145.2
8.	AB461419	USA (Indiana)	117.4	117.4	117.3	146.2
9.	AB461420	China (Mongolia)	117.1	117.1	117.0	137.9

## Data Availability

The nucleotide sequence data used for the calculations are available in GenBank.
